# *Panicum decompositum*, an Australian Native Grass, Has Strong Potential as a Novel Grain in the Modern Food Market

**DOI:** 10.3390/foods12102048

**Published:** 2023-05-18

**Authors:** Jenifer Jenifer, Tina L. Bell, Ali Khoddami, Angela L. Pattison

**Affiliations:** School of Life and Environmental Sciences, Faculty of Science, The University of Sydney, Sydney, NSW 2006, Australia

**Keywords:** Native Millet, wholemeal flour, milling, baking properties, sensory analysis

## Abstract

Native Millet (*Panicum decompositum*) is a native grass species that was used as a staple food by many Australian Aboriginal communities. In this study, the potential for using Native Millet (NM) as a novel flour in the modern food market was investigated. Intact grain and white and wholemeal flours from two populations of NM were compared to bread wheat cv. Spitfire (SW) using a range of physical and chemical tests. The baking properties of NM flour were assessed using basic flatbreads made with 25:75 and 50:50 (NM:SW) mixes of wholemeal flour with 100% SW wholemeal flour used as the control. The grain size of NM was found to be smaller than SW. Milling yield, defined as the proportion of flour obtained from a whole seed, for NM was 4–10% lower than SW under the same moisture conditions used for tempering (drying) wheat. The properties of wholemeal flour indicated that NM flour has lower viscosity and low flour pasting ability compared to SW. This is likely due to the low starch content and high fibre content of NM seed. Wholemeal flour derived from NM had a protein content of 13.6% compared to 12.1% for SW. Based on a sensory analysis using an untrained panel, the distinct colour and texture may negatively affect the acceptance of NM flour by the consumer, but taste and aroma was not found to differ among samples. There were strong indications that the novelty of NM flour may help outweigh any limitations to consumer acceptance, making it a valuable product in future food markets.

## 1. Introduction

For thousands of years, native grasses have been a high carbohydrate staple food for many Australian Aboriginal communities [1]. Seeds from several grass species were consumed raw or transformed into flour to make damper [2,3,4], qualifying it as an ‘ancient grain’. Along with the grain being an important food source for many Aboriginal communities, the harvesting and processing of seed from native grasses has strong cultural significance [5]. The consumption of grass seed has diminished since the colonisation of Australia for a multitude of reasons, one of which being the introduction of alternative grains, such as wheat and barley, alongside the expansion of modern agricultural practices [6,7,8]. In the current market, wheat is the leading cereal crop for flour-based foods across the world [9].

Interest in the use of native grasses in an agriculture setting has increased over the past few decades but the possible use for food production in modern markets and consumer acceptance of these products is unknown. As the food industry is driven by movements towards products with descriptors including ‘ancient’, ‘novel’, ‘sustainable’, and ‘back-to-nature’, the potential for introducing products containing native plant species is high. The full or partial substitution of flour derived from current major cereals with native grass species into human food production is of interest to multiple industries and research disciplines. This is not only because native grasses are well adapted to grow in local environments and are thus likely to be environmentally beneficial but also because other closely related grass species have nutritional benefits and are gluten-free (e.g., *Panicum miliaceum*) [10,11,12]. Furthermore, there is a significant cultural value to Australian Aboriginal people in the revitalisation of native grain systems and a strong desire to reconnect these ancient grains and associated cultural practices to the modern food industry [1].

One of the most common native Australian species of grass used for food by Aboriginal people was *Panicum decompositum* or Native Millet (NM). This perennial species is found throughout mainland Australia and produces flowers and branching seed heads (20–40 cm) in summer and autumn. In arid climates, it flowers in response to rainfall [13]. Nutritionally, a related but non-native grass species, *P*. *miliaceum* (Proso), has a comparable fatty acid composition to wheat and barley [14]. Similarly, *P*. *sumatrense* (Little Millet) is a rich source of phenolic antioxidants [15].

The research described here was carried out in order to evaluate the physical and chemical attributes of seed and flour of NM in comparison to wheat. Such a study is timely, as there is increasing interest in the use of native species worldwide as consumer preference moves towards incorporation of native foods that are putatively more sustainable and nutritious. In Australia, native crops including finger lime, quandong, and lemon myrtle are now widely accepted and used by consumers. According to standardized characterisation of cereal grains, we measured the size, weight, and moisture content of seed and the milling yield (the proportion of flour obtained from a whole grain/seed) at three tempering conditions and the resulting colour of white flour. After recombing white flour with bran to form wholemeal flour, chemical properties, including ash and nitrogen content (as a proxy for protein content) and gelatinisation and pasting quality, were measured or calculated. Furthermore, this research investigated the feasibility of utilising flour from NM as a partial substitute for wheat flour in baking and its acceptance by consumers. To the best of our knowledge, this study represents the first time that seed and flour derived from a native Australian grass has been reported in a peer-reviewed arena.

## 2. Materials and Methods

### 2.1. Seed Preparation and Physical Analysis of Grain

Seed from NM was sourced (machine harvested in 2016) from two wild populations in northern and north-western New South Wales, Australia, located near Quirindi (hereafter referred to as NMQ) (31°30′29″ S, 150°40′48″ E) and Werris Creek (NMW) (31°19′60″ S, 150°39′0″ E). The seed of NM is not produced commercially and can only be harvested in small quantities from natural populations. Seed was hand cleaned by threshing on a rubber board and via vacuum separation to remove chaff, husks, and dust. This process was repeated three to four times until the raw harvested seed was converted into food-grade grain. Once cleaned, NM grain was stored at room temperature and ambient humidity until required.

Grain from bread wheat cv. Spitfire (SW) was used as the bread-making standard in this study. Spitfire is classified as an Australian Prime Hard cultivar and can be sold at a premium to millers both domestically and internationally [16]. Bulk quantities of SW were grown at the IA Watson Grains Research Institute, Narrabri, NSW, Australia in 2016 and stored in a cool room with controlled humidity until required. Wheat grains were pre-cleaned and prepared to use for milling, testing, and baking.

Grain size (width and length, *n* = 3) was measured from images of both types of grains arranged alongside the two rulers positioned at a 90° angle. Accuracy was limited using this method (i.e., to the nearest mm), so data were only used for general comparison among grains. Grain weight was measured using the 1000-kernel weight (TKW) method [17]. The manual counting of 100 grains was performed in triplicate and the weight was converted into TKW (g). Test weight (kg hL^−1^; *n* = 5), used as an indication of soundness of grain and correlated with flour yield, was measured according to American Association of Cereal Chemists (AACC), Method 55-10.01 [18].

The moisture content of grain was determined for milling purposes. Duplicate samples of approximately 3 g of seed were weighed, dried at 130 °C for 1 h to constant weight, and re-weighed (AACC Method 44-01.01) [18]. For the two samples of NM from different populations, the moisture content of grain was greater than recommended for milling. To correct this, approximately 650–700 g of seed from each population was dehydrated at 40 °C overnight and the moisture content was remeasured using the same method.

### 2.2. Milling

White flour was used to determine milling yield and flour colour [19]. For the production of white flours, the duplicate samples of grains were tempered to 11.5, 13.5, and 15.5% moisture. These levels were selected based on common milling moisture content by AACC Method 26-10.02 [18]. Samples were tempered by weighing approximately 100 g of grain and adding the required amount of water based on the initial moisture content and the final moisture desired. After the addition of water, containers of grain were placed on an automatic shaker for 15 min then left to stand overnight to equilibrate. Tempered grain was milled to white flour (Quadrumat Junior Mill, Brabender, Duisburg, Germany) using identical settings for both SW and NM. Milling yield (%) was calculated as the ratio of white flour to initial grain weight.

Wholemeal flour was used to examine the selected chemical and baking properties of SW compared to NM and for the preparation of flatbreads for sensory analysis. Wholemeal flour was produced using a hammer mill (Newport Scientific 600, Warriewood, Australia) fitted with a 0.8 mm screen and run at a low feeder speed. Unless otherwise specified, three replicate samples of white or wholemeal flour from NM and SW were analysed.

### 2.3. Chemical and Physical Analysis of Flour

The colour of white flour was measured using a CR-300 Minolta Chroma Meter (Minolta Co., Ltd., Osaka 541, Japan). The colour measurements were quantified via the Hunterlab system giving values for L*, a*, and b* parameters. Maximum L* is 100 and represents white, and minimum L* is zero and represents black. The values for a* and b* have no specific numerical limits; however, positive a* is red, negative a* is green, positive b* is yellow, and negative b* is blue [20].

The nitrogen content (%N) of the duplicate samples of wholemeal flour was measured using an element analyser (Elementar Vario Max CNS Analysensysteme GmbH, Hanau, Germany). Nitrogen content was converted to protein content using the universal conversion factor of 6.25 [21].

The ash content of the duplicate samples of wholemeal flour was determined using the method described by Khoddami et al. [22]. Samples (approximately 0.5 g) were weighed in porcelain crucibles and transferred into a muffle furnace and heated at 550 °C until a constant weight was reached. The ash content was calculated and expressed as a percentage (%) of wet weight.

The gelatinisation and pasting quality of wholemeal flour was tested using a standard Rapid Visco Analyser (RVA) (Newport Scientific, Warriewood, Australia) according to AACC Method 76-21.02 [18]. This method was used to measure the physiochemical and functional properties of starch in the flour when heated and cooled and of the final gelatinised product [20,23]. The results can be used to describe the texture of the final product [24].

### 2.4. Preparation and Quality Testing of Flatbread

Flatbreads were prepared using wholemeal flour, salt, and water. Pure wholemeal flour from NM grain was found to be unsuitable for bread-making purposes so the partial substitution of wheat was chosen for product quality testing and sensory analysis. The three formulations used were: 100% SW flour (100 SW), a 25:75 mix of NM and SW flours (25:75 NM:SW), and a 50:50 mix of NM and SW flours (50:50 NM:SW). Given the overall similarity of wholemeal flours produced from grain collected from the two populations of NM (NMQ and NMW; see Section 3) and the limited amount of seed and flour available, both samples of wholemeal NM flour were combined in equal proportions to make flatbreads. The amount of water required for each dough was determined through the measurement of flour water absorption [25]. For this method, the amount of water is subjectively measured through the formation of a dough that is non-sticky, smooth, and appropriate for sheeting. Doughs made from 100 SW, 25:75 NM:SW, and 50:50 NM:SW required 167, 161, and 168 mL water, respectively.

The method for the preparation of dough was adapted from Kahlon and Chiu [26]. In a large bowl, salt (1.7 g) and wholemeal flour (250 g) were combined, and the dough was formed by adding water gradually using a stand mixer with a dough hook attachment (Model B7C, FSM, Prestons, Australia). After mixing for 5 min, a smooth and non-sticky dough was formed. The dough was rested at room temperature for 30 min. The rested dough was divided into 60 g portions (*n* = 7) and flattened between two sheets of plastic wrap by hand and a rolling pin with a 2 mm thickness guide attached.

The appropriate cooking temperature was determined through the trial baking of flatbreads made from commercial wheat flour at 250 °C (3 min) and at 200 °C (5 min) using a 10 Tray Plus fan-forced Electric Combi Oven (Unox, Cadoneghe, Italy). It was decided that 200 °C for 5 min produced a flatbread with the best consistency. The flatbreads were cooled at room temperature for 1 h and stored at room temperature in polyethylene zip-lock bags for further analysis.

Flatbreads were analysed using several quality measures, including the colour of the baked product and moisture loss after baking and after 7 days of storage. The colour of the cooked flatbread was measured at five random spots on each surface. The L*, a*, and b* values were recorded using a portable colorimeter (Chroma Meter CR-400, Minolta Camera Co., Osaka, Japan). The loss of moisture (%) from the flatbreads after 7 days of storage was measured by placing three individual wedges from each type of flatbread in separate semi-permeable polyethylene zip-lock bags. Initial weight was taken for each sample and again after 7 days of storage at room temperature.

### 2.5. Sensory Analysis of Flatbread

The sensory analysis method was adopted from Singh et al., Adebiyi et al., and Alencar et al. [27,28,29]. Sensory analysis trials were held in the Food Analysis Laboratory at the University of Sydney, Australia. A controlled environment was used to ensure that environmental conditions were both safe and repeatable for the panellists. Volunteer untrained panellists (*n* = 50) were briefed prior to testing to confirm that they had no food allergies. A project information sheet, consent form, and sensory analysis questionnaire were provided to each panellist before the sensory test. Panellists were asked to evaluate each type of flatbread according to appearance, colour, flavour, aroma, texture, and overall acceptability using a 1 to 7 hedonic scale for each attribute (see Supplementary Information). Panellists were also provided with the option to add additional comments. Samples (one wedge of each type of wholemeal flatbread) were presented in a random order on a white rectangular plate labelled with a three-digit randomly generated number. Water was provided for palate cleansing before and between each sample test. The sensory analysis was causal, and a fasting mouth was not required. This approach was taken to determine how the general consumer would response to a novel product instead of a specialised trained panel. The sensory testing lasted for approximately 20 min for each panellist. Sensory trials were approved by the University of Sydney Human Ethics Committee, reference number 2018/074.

### 2.6. Statistical Analysis

Data were analysed through a one-way analysis of variance (ANOVA) using IBM SPSS statistics 27. Grain and flour properties and properties of flatbread were compared using a significance level of *p* < 0.05.

## 3. Results

### 3.1. Physical Properties of Whole Grain

**Figure 1:**
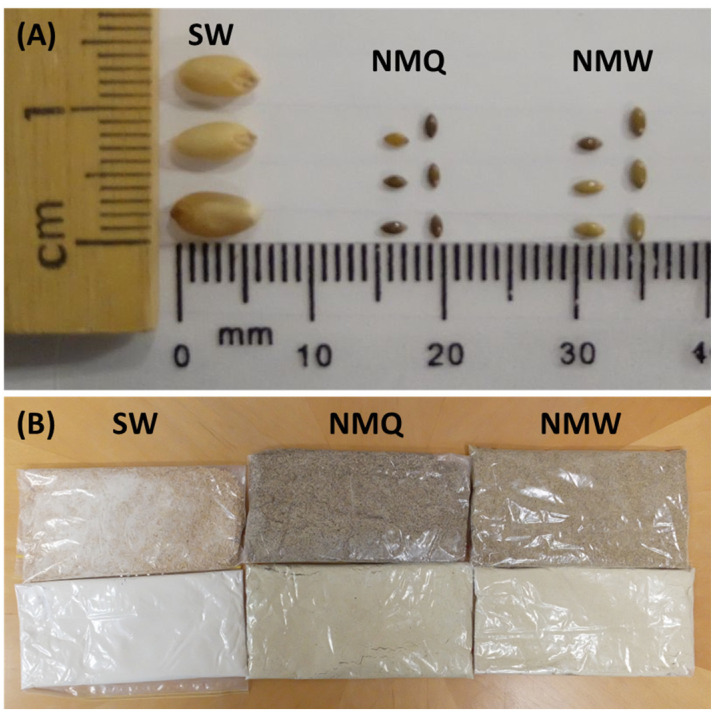
Differences in (**A**) seed size and (**B**) flour colour of grain of bread wheat cv. Spitfire (SW) and Native Millet (*Panicum decompositum*) harvested from populations located near Quirindi (NMQ) and Werris Creek (NMW). For flour samples (**B**), wholemeal flour is represented by three samples in the top row and white flour is represented by the three samples in the bottom row.

The grain of NM harvested from both populations (Quirindi and Werris Creek) were smaller (e.g., length and width) and lighter (e.g., TKW) than SW (Table 1, Figure 1A). The test weights of NMQ, NMW, and SW had a small range (i.e., 76.7–80.5 kg hL^−1^), but the grain of SW was significantly heavier. The grain of NM had significantly higher moisture content than SW but was not significantly different between the two populations (Table 1).

### 3.2. Milling Yield

**Figure 2:**
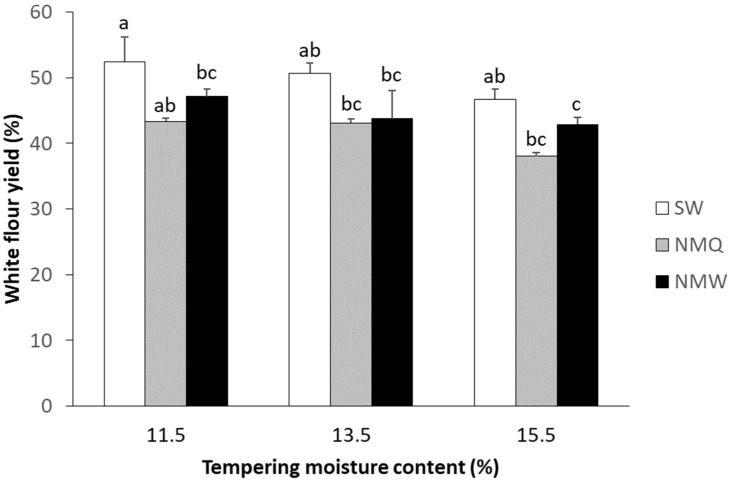
Milling yield for white flour from grain of bread wheat cv. Spitfire (SW) and Native Millet (*Panicum decompositum*) harvested from populations located near Quirindi (NMQ) and Werris Creek (NMW) at different tempering moistures. Bars represent mean ± standard deviation (*n* = 2). Different letters indicate significant differences among grain type and tempering moisture content at *p* < 0.05.

White flour yield was higher for SW compared to both NMQ and NMW for all tempering moisture contents tested (Figure 2). The highest milling yield for all grain types was achieved at a tempering moisture content of 11.5%, with reduced yield at higher tempering moisture content. Overall, the difference in white flour yield among SW and NM collected from two different populations was significant (ranging from 4–9%; one-way ANOVA, *p* = 0.002), and tempering moisture had significant effect on flour yield (one-way ANOVA, *p* = 0.008). For a given grain type, tempering moisture content had little influence on white flour yield except for NMQ grain tempered at 15.5% moisture content, which had significantly lower yields compared to 11.5 and 13.5% moisture content. Overall, milling yield for NMW was up to 4% higher than NMQ, but these differences were not significant. There were no observable differences during the milling process for SW compared to NM (e.g., no adjustments were need for the smaller seed size of NM).

### 3.3. Physical and Chemical Properties of Flour

Protein and ash content of wholemeal flour made from NM grain was higher compared to SW (Table 2). Both measures of flour quality were significantly different for SW compared to NMQ and NMW (one-way ANOVA, *p* < 0.001). For all the physical and chemical properties measured for grain and flour, the ash content of wholemeal flour made from the two populations of NM was the only measure that differed significantly (Table 2).

There were obvious observable differences in colour among white flours milled from SW, NMQ, and NMW (Figure 1B). In general, NM flours were darker (i.e., lower L*) and greener (i.e., negative values of a*) and yellower (i.e., higher b*) compared to white flour from SW (Table 3). For the two populations of NM, white flour produced from grain from Werris Creek has a lighter and less greenish colour than white flour from grain from Quirindi. The differences in flour colour among grain types was significant (one-way ANOVA, *p* < 0.001), and the effect of adding water for the purpose of tempering the grains was significant for both L* and a* values (one-way ANOVA, *p* < 0.05) but not for b*. In contrast, the colour of flour from grain collected from two populations of NM were generally similar among tempering moisture content (e.g., L* and b* values; Table 2).

The amylographic assessment of wholemeal flour using RVA indicated that NM flour samples had significantly lower measures of peak, trough, breakdown, setback, peak time, and final viscosity compared to SW flour (one-way ANOVA, *p* < 0.001; Table 4). Overall, the gelatinisation and pasting parameters of NMQ and NMW flours were not statistically different.

### 3.4. Quality of Flatbreads

**Figure 3:**
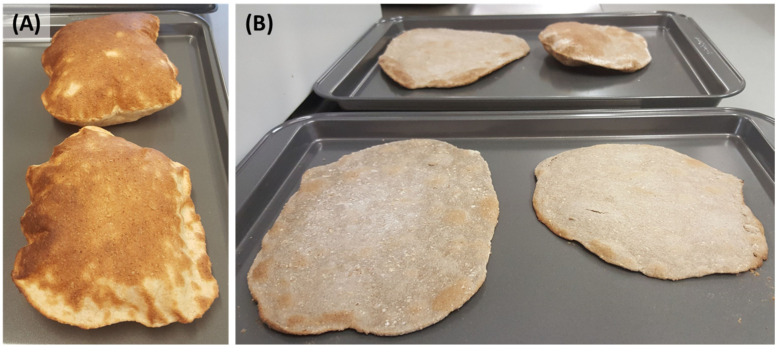
Representative flatbreads made from (**A**) 100% flour from bread wheat cv. Spitfire (100 SW) and ((**B**), upper two samples) a mix of flours at 50% NM and 50% SW (50:50 NM:SW) and (lower two samples) a mix of flours at 25% Native Millet and 75% SW (25:75 NM:SW).

Flatbreads made from 100 SW appeared puffed and golden after baking (Figure 3A) compared to flatbreads containing 50:50 NM:SW (Figure 3B, lower two samples). Flatbreads with a smaller proportion of NM flour (i.e., 25:75 NM:SW) showed some capacity to rise (Figure 3B, upper two samples).

As expected, moisture was lost during the baking of flatbreads. However, flatbreads made from 50:50 NM:SW lost significantly more moisture during baking (36.1 ± 3.3%) (one-way ANOVA, *p* < 0.001) compared to flatbreads made from 100 SW (25.6 ± 3.1%) and 25:75 NM:SW (25.5 ± 3.9%). In comparison, moisture losses from all flatbreads after 7 days of storage were small and were not statistically different (range of 1.1 to 1.2%).

The characteristic green/yellow colour of NM wholemeal flour remained evident after baking, as indicated by significantly low a* and high b* values for flatbreads made with both 25:75 NM:SW and 50:50 NM:SW flour mixes (one-way ANOVA, *p* < 0.001). Colour changes (browning) occurred on both top and bottom surfaces of all samples during the baking process. Overall, there was a significant difference in browning on top surfaces according to an increase in reddish and yellowish colour (i.e., high a* and b*) (one-way ANOVA, *p* < 0.001), and a darker colour developed on the bottom surface (i.e., low L*) (Table 5).

### 3.5. Sensory Analysis of Flatbreads

**Figure 4:**
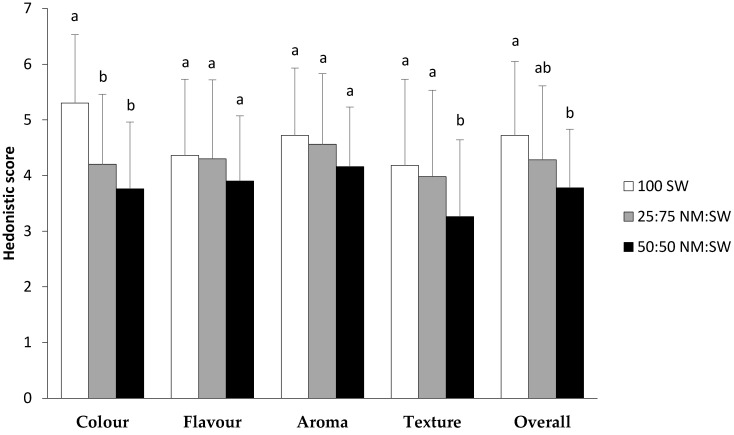
Sensory scores for flatbreads for a range of characters and an overall score. Values presented are mean ± standard deviation (*n* = 50). Different letters indicate significant differences within each character at *p* < 0.05. 100 SW: 100% flour from bread wheat cv. Spitfire (SW); 25:75 NM:SW: mix of flours at 25% Native Millet (NM) and 75% SW; 50:50 NM:SW: mix of flours at 50% NM and 50% SW. The Hedonistic score used was 1: dislike very much; 2: dislike; 3: dislike slightly; 4: neutral; 5: like slightly; 6: like; 7: like very much.

Overall, the sensory analysis of the three types of flatbreads indicated that, for most of the aspects tested, flatbreads made from SW were preferred in comparison to flatbreads made from a mix of flours (Figure 4). Flatbreads made from SW flour were preferred by panellists in terms of colour but differences in aroma and flavour among samples were not significantly different. The only exception was for texture, where flatbread made with a 50:50 NM:SW mix was preferred (Figure 4).

Free-text comments made by some participants (more than 50% of the cohort) reflected different baking properties of NM flour with the crisper nature of flatbreads made with 50:50 NM:SW, a feature that was both liked and disliked. In addition, some participants noted a grainy or sandy quality to flatbreads containing NM flour, which is very likely to have influenced their scoring pattern. As indicated by the Likert scale, colour was important, and this was confirmed with several participants commenting on the unappealing colour of flatbreads containing NM flour. Few participants commented on aroma but, when they did, they liked the aroma of flatbread with NM flour, particularly after chewing the baked product. Comments regarding flavour or taste of flatbreads with NM flour were mixed with an equal number of responses indicating that the flavour was appealing or unappealing.

## 4. Discussion

This study investigated a selection of processing, baking, and sensory properties of NM compared to a cultivar of wheat (SW). We found that, although high in protein compared to wheat, white flour yield was lower. The traditional food made by Aboriginal people from NM was an unleavened bread cooked over hot coals and consumed immediately [1]. The physicochemical properties of wholemeal NM flour suggest that recreating baked products resembling how it was used traditionally is untenable [1]. Such a product would be difficult to process mechanically as the dough does not hold together and would lack modern consumer appeal. When wholemeal NM flour was combined with wheat flour and used to make flatbreads, sensory analysis by an untrained volunteer panel indicated mixed appeal of products containing even a small proportion (i.e., 25%) of NM. Changes in cooking methods, food quality standards, and consumer expectations have created a need to understand NM flour in a modern context. Products made from 100% chia or quinoa flour had a similar perception in the modern food industry [30,31]; however, following research, these ancient grains have gained greater acceptance when used in different forms.

### 4.1. Influence of Grain Size and Chemistry on Flour Properties

The small grain size of NM relative to SW resulted in a greater proportion of bran to endosperm. This was reflected in ash content, milling yields, colour, and pasting and gelatinisation profiles. The darker colour of NM flour was partially due to the colour of the bran. The inner husk of NM is usually dark brown and shiny and is difficult to remove [5]. As it was traditionally consumed [32] and likely to be included in modern foods made from NM, it was appropriate to allow this part of the husk to be included in our research. Whilst most of the husk would have been included in the bran portion during milling, pieces of this dark husk within the white flour portion would have impacted the flour colour, causing lower L* values (brightness/whiteness) compared to white flour from SW. However, rather than being interpreted solely as differences in bran content, lower L* may be due to the inherent colour of the endosperm and a range of other chemical and physical properties [19,33,34]. This suggests that further testing of chemical (e.g., phytochemical content and antioxidant activity) and physical properties (e.g., particle size) of white and wholemeal flour from NM is warranted. This information is important as flour colour often determines end-product use and market price [35].

The RVA profiles assisted to provide more information on product texture. Lower peak, and final viscosities for both NM flours compared to SW flour could be an indication of a lower starch content or a different amylose/amylopectin ratio, which leads to a weak dough that produces a food product with an inferior texture [24]. In addition to a low proportion of starch, the lower peak viscosity of NM flour may be influenced by a lack of gluten, lower content of water-absorbing non-starch polysaccharides such as arabinoxylans and other pentosans, or it might contain great level of protein or sugar digesting enzymes [36]. However, the inclusion of the inner husk, which is likely to be high in cellulose and insoluble fibre, is another likely influence on low pasting viscosity.

Gluten is an important storage protein in wheat flour and, arguably, its presence or absence has the greatest impact on the baking quality of any type of flour [37,38]. Flours that do not contain gluten must be treated or used differently compared to flours that contain gluten. It is unlikely that NM produces glutenins, the precursor to gluten, due to the genetic divergence of ancestral grasses and related species from domesticated wheat [39]. As a putative indication of good dough production and bread quality, the protein content of wholemeal flour made from NM was higher than from SW. However, flatbread made from 50:50 NM:SW lost the most moisture during baking and was noted as being crisp during sensory analysis in comparison to 100 SW and 25:75 NM:SW flatbreads.

### 4.2. Use of Wholemeal versus White Flour from Native Millet

Post-harvest production costs are a major contributor to the profitability of wheat processing and are usually related to milling yield and quality [40]. Both SW and NM produced lower milling yields than the standard commercial minimum (68%) [41]. Such low yields could be due to the use of a Junior Mill, which has fewer rollers and lower efficiency than a standard commercial mill, thus the results are not readily comparable to commercial white flour regardless of grain type. The range of grain tempering conditions used in this study were within the range suitable for wheat and, with additional testing varying moisture content and equilibration time, the conditions to maximise flour yield will be optimized [19,42]. Nevertheless, the proportion of bran in NM was significantly higher than SW (up to 60%) and the value of producing ‘white’ flour from NM, both economic and otherwise, should be carefully considered by the food industry. By blending wholemeal NM flour with wheat or other flours in the final product, the disadvantages of wholemeal NM flour on product quality can be minimised whilst maintaining its nutritional benefits, plus without losing approximately 50% of the NM grain weight (with its associated economic costs) during processing.

### 4.3. Consumer Acceptance of Native Millet Flour

This research suggests further advantages to using wholemeal NM flour blends as opposed to pure NM flour. This study used the relatively high proportions of NM flour (25 and 50%) to create two composite flours for baking. Studies that have used similar ratios of novel flour in composite mixes found greatest consumer acceptability at a substation rate of 25% (e.g., wholegrain barley flour) [43] or higher (e.g., 60% substitution using wholegrain rye or barley flour or oat flake meal) [44] (70% substitution using barley flour) [45]. At the highest end of the scale, breads made from 100% wholegrain flour from rye, oat, sorghum, and millet had acceptable sensory properties, particularly for appearance, crumb, and the development of pores, although flours were thermally and hydrothermally modified prior to baking [46]. Other studies have used far lower rates of substitution of flour from novel sources and, while functional properties of end products can remain similar compared to 100% wheat flour (e.g., 10% soy flour or 15% barley flour) [47], consumer acceptability due to colour and flavour may be low (e.g., 5% substitution using banana, pumpkin, and mango flour) [48]. While these examples indicate that consumer acceptability of the end-product varied with flour type, all studies were introduced with the notion of using novel flour to improve health benefits of flour-based end products.

Given the poor pasting properties, unappealing colour, small seed size, and potentially high cost of NM flour, future research should investigate a smaller proportion of NM in composite flour mixes (e.g., 15%) to determine whether baked products are more appealing to consumers and, potentially, making them more affordable. Furthermore, baked products that are better suited to NM flour than flatbread should be investigated. According to the sensory analysis, composite NM flour flatbreads had a dry granular texture, unappealing colour, and slight aftertaste. However, panellists noted that these flatbreads were crispy and more like a cracker than a flatbread, indicating a possible alternative use of this grain. Flour derived from NM grain had a dark green-yellow colour compared to SW flour, making it potentially less marketable if substituted into end products that are expected to be a particular colour. End-products are less likely to be accepted or liked by consumers if the colour does not match with their emotional expectations [49].

The use of seed from NM and other Australian native grasses has four broad benefits: health benefits [50], environmental impact [51], economic stimulation in rural and remote areas [52], and deep connections to Aboriginal culture [1]. Consumer preference is changing and having healthier food sources with lesser impact on the environment or that provide social benefits is becoming more acceptable. In this study, panellists indicated that knowing the broad benefits of NM prior to the sensory analysis may have influenced their assessment of flatbreads. Pre-knowledge of the uniqueness of NM flour may outweigh the importance of colour, texture, and flavour of a baked product to a point where they may not be considered negative qualities. The anecdotal evidence provided by our sensory analysis revealed huge potential for use of seed from native grasses as new food products and will be valuable in designing subsequent baking and sensory tests for NM and other species.

From an environmental viewpoint, native grasses require minimal additional nutrient and water inputs compared to agricultural cereals [53] and can be beneficial in maintaining soil health [54]. In the current market, the standard of milling-grade wheat is usually 12% protein based on whole-grain measures. Nitrogen fertiliser is required to increase the grain protein content of wheat, and under most conditions, growers invest their money in just enough fertilisers to reach the benchmark of 12% [40]. Thus, NM can potentially be marketed as grain for its high protein content even when no fertiliser has been added, resulting better outcomes for the environment.

Arguably the most compelling issue facing the use of NM grain in a modern food context is the proper recognition of the cultural importance of exploitation of any species of native flora or fauna or other natural resource [55]. One key element for developing the use of native grains in the modern food market is to establish appropriate and bespoke practices, including accounting for culture, knowledge, and heritage asset value for equitable return of any economic royalties or profits to Indigenous communities [56]. Involving Aboriginal people in the development of the new processes and foods which use NM early in the product development cycle will help achieve this. As the consumer acceptance of NM foods is likely to be linked to its connections to Aboriginal culture, the most valuable research will partner industry with Aboriginal custodians of traditional grain knowledge. This would be a divergence from typical food research and requires cross-disciplinary and multi-faceted cooperation. Recent interest of consumers in healthier food options with higher dietary fibre (e.g., inclusion of wheat bran) is a good example of diverging from a more-or-less linear progression of grain-quality innovation [57]. The best progression for the incorporation of native grains into the modern food market is unlikely to be linear, as combining the knowledge of Aboriginal people, food technologists, and food scientists in a respectful and inclusive way may lead to the greatest innovation and value for all.

## 5. Conclusions

The broad cultural, environmental, economic, and human health benefits associated with use of NM in Australia have driven the need to understand how this ancient grain can be incorporated into modern foods. From the various components of this study, it can be concluded that there is a potential for the inclusion of Australian Native Millet (NM) in certain markets and in certain food products. This market can be reached by using wholemeal flour to reduce costs, blending flour to balance sensory acceptance with nutritional benefits, and ensuring the rich cultural connections of NM are maintained by creating equal and respectful partnerships between Aboriginal people, the food industry, and cross-disciplinary research. Further investigation into uses of NM in a variety of products at various blend ratios and the consumer acceptance of these products are recommended. Native Millet is only one example of a grass species that was traditionally used by Indigenous Aboriginals, so future research should also investigate the potential of other native species, including their ecophysiological responses to environmental conditions and how grain production and composition may be affected.

## Figures and Tables

**Table 1:** Physical properties of grain of bread wheat cv. Spitfire (SW) and Native Millet harvested from populations located near Quirindi (NMQ) and Werris Creek (NMW). Values presented are mean ± standard deviation (*n* = 3 for grain length/width and TKW; *n* = 5 for test weight; *n* = 2 for moisture content). Means within a column with different letters are significantly different at *p* < 0.05. TKW: 1000-kernel weight.

Grain	Grain Length/Width (mm)	TKW (g)	Test Weight (kg hL^−1^)	Moisture Content (%)
SW	6/4	38.6 ± 1.2 ^a^	80.5 ± 0.3 ^a^	9.5 ± 0.02 ^a^
NMQ	2/1	0.9 ± 0.1 ^b^	76.7 ± 1.3 ^b^	13.2 ± 0.01 ^b^
NMW	2/1	1.2 ± 0.1 ^b^	77.3 ± 0.4 ^b^	13.5 ± 0.06 ^b^

**Table 2:** Chemical properties of wholemeal flour milled from grain from bread wheat cv. Spitfire (SW) and Native Millet (*Panicum decompositum*) harvested from populations near Quirindi (NMQ) and Werris Creek (NMW). Values presented are mean ± standard deviation (*n* = 2). Means within a column with different letters are significantly different at *p* < 0.05.

Flour	Protein (%)	Ash (%)
SW	12.1 ± 0.2 ^b^	1.6 ± 0.01 ^c^
NMQ	13.7 ± 0.4 ^a^	6.7 ± 0.06 ^b^
NMW	13.6 ± 0.1 ^a^	7.2 ± 0.01 ^a^

**Table 3:** Colour properties of white flour from grain from bread wheat cv. Spitfire (SW) and Native Millet (*Panicum decompositum*) harvested from populations located near Quirindi (NMQ) and Werris Creek (NMW). The grains were tempered at three different moisture contents prior to milling. Data are presented as mean ± standard deviation (*n* = 2). Means within a column with different letters are significantly different at *p* < 0.05.

Flour	Moisture Content (%)	L* Value	a* Value	b* Value
SW	11.5	91.81 ± 0.20 ^a^	0.16 ± 0.08 ^ab^	7.74 ± 0.19 ^b^
	13.5	91.31 ± 1.27 ^a^	0.14 ± 0.01 ^abc^	7.57 ± 0.14 ^b^
	15.5	92.50 ± 0.23 ^a^	0.06 ± 0.06 ^abc^	7.74 ± 0.27 ^b^
NMQ	11.5	76.24 ± 0.38 ^d^	0.25 ± 0.12 ^ab^	17.76 ± 0.12 ^a^
	13.5	77.75 ± 0.23 ^cd^	-0.14 ± 0.12 ^bcd^	17.84 ± 0.26 ^a^
	15.5	79.27 ± 0.52 ^bc^	-0.60 ± 0.08 ^d^	18.14 ± 0.01 ^a^
NMW	11.5	78.57 ± 1.37 ^bcd^	0.28 ± 0.23 ^ab^	17.52 ± 0.22 ^a^
	13.5	77.87 ± 0.98 ^bcd^	0.48 ± 0.16 ^a^	17.56 ± 0.76 ^a^
	15.5	80.86 ± 0.30 ^b^	-0.34 ± 0.06 ^cd^	18.05 ± 0.07 ^a^

**Table 4:** Rapid visco analysis (RVA) of wholemeal flour milled from grain from bread wheat cv. Spitfire (SW) and Native Millet (*Panicum decompositum*) harvested from populations near Quirindi (NMQ) and Werris Creek (NMW). Values for viscosity are reported as RVA units and presented as mean ± standard deviation (*n* = 3). Means within a column with different letters are significantly different at *p* < 0.05.

Flour	Peak Viscosity cP	Holding Strength cP	Breakdown cP	Setback cP	Final Viscosity cP	Peak Time (min)
SW	195.92 ± 3.25 ^a^	112.17 ± 5.21 ^a^	83.75 ± 2.34 ^a^	113.16 ± 2.51 ^a^	225.33 ± 4.46 ^a^	5.62 ± 0.04 ^a^
NMQ	58.32 ± 1.40 ^b^	43.53 ± 1.07 ^b^	14.78 ± 0.38 ^b^	71.58 ± 2.21 ^b^	115.11 ± 3.20 ^b^	5.35 ± 0.04 ^b^
NMW	53.97 ± 1.00 ^b^	40.47 ± 0.93 ^b^	13.50 ± 0.30 ^b^	67.69 ± 1.96 ^b^	108.16 ± 2.77 ^b^	5.33 ± 0.07 ^b^

**Table 5:** Colour properties post-baking of flatbreads made from bread wheat cv. Spitfire (SW) and Native Millet (NM). Values presented are mean ± standard deviation. Means within a column with different letters are significantly different at *p* < 0.05. 100 SW:100% flour from bread wheat cv. Spitfire; 25:75 NM:SW: mix of flours at 25% Native Millet and 75% SW; 50:50 NM:SW: mix of flours at 50% NM and 50% SW.

Flour Mix	L* Value	a* Value	b* Value
Top of flatbread			
100 SW	55.42 ± 1.63 ^a^	4.91 ± 0.55 ^a^	16.19 ± 0.47 ^a^
25:75 NM:SW	49.21 ± 1.90 ^c^	3.58 ± 0.50 ^b^	12.23 ± 0.34 ^b^
50:50 NM:SW	52.75 ± 1.76 ^b^	2.35 ± 0.24 ^c^	11.21 ± 0.85 ^c^
Bottom of flatbread			
100 SW	58.07 ± 2.26 ^a^	4.12 ± 0.21 ^a^	16.63 ± 0.70 ^a^
25:75 NM:SW	45.80 ± 1.73 ^b^	3.66 ± 0.19 ^b^	11.39 ± 0.56 ^b^
50:50 NM:SW	42.57 ± 1.85 ^c^	3.45 ± 0.18 ^c^	9.55 ± 0.72 ^c^

## Data Availability

The data presented in this study may be made available on request from the corresponding author. The survey data are not available due to restrictions applied by Human Ethics, the University of Sydney, for the protection of privacy.

## Supplementary Materials

The following supporting information can be downloaded at: https://www.mdpi.com/article/10.3390/foods12102048/s1. Hedonic scale test.
