# Supplementary material for: Panicum decompositum, an Australian Native Grass, Has Strong Potential as a Novel Grain in the Modern Food Market
Source: Foods. 2023 May 18;12(10):2048. doi: 10.3390/foods12102048 (PMC10217037; doi:10.3390/foods12102048)
Supplement: Supplementary file 1 [file foods-12-02048-s001.zip › foods-2358062-supplementary.pdf]

Supplementary information

Hedonic scale test

Panellist number: ____________________
Date: ______________________________

There are three flatbreads sample provided in front of you, please rate each sample for the attributes listed below.

To do this test, please write down the sample code in the box provided and taste each sample from left to right. Rinse your mouth with water between each sample.

Thank you for your participation.

Scale:
1 – Dislike very much
2 – Dislike
3 – Dislike slightly
4 – Neutral
5 – Like slightly
6 - Like
7 – Like very much

Sample code: [ ]

Indicate the difference level on scale line below:

	**1**	**2**	**3**	**4**	**5**	**6**	**7**
Colour	○	○	○	○	○	○	○
Flavour	○	○	○	○	○	○	○
Aroma	○	○	○	○	○	○	○
Texture	○	○	○	○	○	○	○
Overall	○	○	○	○	○	○	○

Sample code:

Indicate the difference level on scale line below:

	1	2	3	4	5	6	7
Colour	○	○	○	○	○	○	○
Flavour	○	○	○	○	○	○	○
Aroma	○	○	○	○	○	○	○
Texture	○	○	○	○	○	○	○
Overall	○	○	○	○	○	○	○

Sample code:

Indicate the difference level on scale line below:

	1	2	3	4	5	6	7
Colour	○	○	○	○	○	○	○
Flavour	○	○	○	○	○	○	○
Aroma	○	○	○	○	○	○	○
Texture	○	○	○	○	○	○	○
Overall	○	○	○	○	○	○	○

Comments:
